# Supplementary material for: A case study of transferring the effect of demographic factors on e-waste recycling to the waste container assignment model
Source: PLoS One. 2025 Aug 25;20(8):e0315695. doi: 10.1371/journal.pone.0315695 (PMC12377600; doi:10.1371/journal.pone.0315695)
Supplement: S1 Table — (PDF) [file pone.0315695.s001.pdf]

S1 Table. Number of houses for each neighborhood (1)

	Neighborhood Names	***Neighborhood Codes***	***Number of Houses***
1	Shukrupasa	*1. Region*	13512
2	Liberation	*2. Region*	15721
3	Omar Nasuhi	*3. Region*	9491
4	Lalapasha	*4. Region*	12714
5	Rabia	*5. Region*	10190
6	Murat pasha	*6. Region*	6635
7	University	*7. Region*	1801
8	Karabekirpasha	*8. Region*	8624

Source: 1. https://www.endeksa.com/tr/analiz/turkiye/erzurum/demografi [Internet].
