# Supplementary material for: A case study of transferring the effect of demographic factors on e-waste recycling to the waste container assignment model
Source: PLoS One. 2025 Aug 25;20(8):e0315695. doi: 10.1371/journal.pone.0315695 (PMC12377600; doi:10.1371/journal.pone.0315695)
Supplement: S2 Table — (PDF) [file pone.0315695.s002.pdf]

**S2 Table. Distances between demand points and candidate container points ($d_{ij}$).**

| | *1. Region* | *2. Region* | *3. Region* | *4. Region* | *5. Region* | *6. Region* | *7. Region* | *8. Region* |
|---|---|---|---|---|---|---|---|---|
| **1** | 960 | 1190 | 2210 | 2730 | 1800 | 2850 | 2990 | 3920 |
| **2** | 1410 | 1590 | 2270 | 2230 | 1210 | 2550 | 3090 | 3990 |
| **3** | 1590 | 1030 | 2740 | 2130 | 1300 | 2190 | 2540 | 3360 |
| **4** | 1980 | 1560 | 2880 | 1630 | 720 | 1920 | 2740 | 3480 |
| **5** | 2530 | 680 | 2850 | 2780 | 2520 | 2210 | 1230 | 2100 |
| **6** | 1560 | 590 | 2930 | 2430 | 1730 | 2300 | 2270 | 3110 |
| **7** | 1910 | 690 | 3220 | 2130 | 1540 | 1950 | 2040 | 2840 |
| **8** | 2280 | 720 | 3620 | 2090 | 1720 | 1720 | 1560 | 2410 |
| **9** | 1180 | 2630 | 910 | 3430 | 2330 | 3880 | 4330 | 5210 |
| **10** | 1670 | 2730 | 1450 | 2930 | 1850 | 3520 | 4310 | 5120 |
| **11** | 1550 | 2140 | 1930 | 2400 | 1300 | 2900 | 3650 | 4470 |
| **12** | 3480 | 2810 | 4040 | 400 | 870 | 1390 | 4150 | 3570 |
| **13** | 3880 | 3040 | 4520 | 300 | 1310 | 1170 | 3140 | 3400 |
| **14** | 4010 | 2960 | 4790 | 480 | 1580 | 780 | 2750 | 2980 |
| **15** | 2200 | 1880 | 2850 | 1490 | 410 | 2010 | 3050 | 3780 |
| **16** | 2800 | 2180 | 3490 | 840 | 400 | 1440 | 2830 | 3420 |
| **17** | 2950 | 2530 | 3460 | 880 | 310 | 1720 | 3210 | 3770 |
| **18** | 3420 | 1940 | 4550 | 1310 | 1740 | 490 | 1570 | 1960 |
| **19** | 3120 | 1820 | 4150 | 1020 | 1290 | 680 | 1860 | 2420 |
| **20** | 3170 | 1360 | 4650 | 2710 | 2740 | 1900 | 530 | 1410 |
| **21** | 3640 | 2200 | 3230 | 1470 | 430 | 2500 | 3470 | 1140 |
| **22** | 4490 | 2650 | 5860 | 2890 | 3370 | 1850 | 830 | 340 |
| **23** | 5130 | 3260 | 6640 | 3770 | 4240 | 2740 | 1360 | 490 |
