# Supplementary material for: A case study of transferring the effect of demographic factors on e-waste recycling to the waste container assignment model
Source: PLoS One. 2025 Aug 25;20(8):e0315695. doi: 10.1371/journal.pone.0315695 (PMC12377600; doi:10.1371/journal.pone.0315695)
Supplement: S3 Table — (PDF) [file pone.0315695.s003.pdf]

S3 Table. Distribution of candidate containers by neighborhood

	Neighborhood	*Candidate Container Numbers*
**1**	1. Region	2
**2**	2. Region	2
**3**	3. Region	4
**4**	4. Region	4
**5**	5. Region	3
**6**	6. Region	3
**7**	7. Region	3
**8**	8. Region	2
	**Total**	**23**
