# Supplementary material for: A case study of transferring the effect of demographic factors on e-waste recycling to the waste container assignment model
Source: PLoS One. 2025 Aug 25;20(8):e0315695. doi: 10.1371/journal.pone.0315695 (PMC12377600; doi:10.1371/journal.pone.0315695)
Supplement: S4 Table — (PDF) [file pone.0315695.s004.pdf]

S4 Table. Population for the selected neighborhoods and the amount of income per capita for each one (1)

	Neighborhood Codes	*Neighborhood Population*	*Per Capita Income (₺)*
**1**	*1. Region*	36.892	2.241
**2**	*2. Region*	32.301	2.067
**3**	*3. Region*	24.338	2.568
**4**	*4. Region*	22.801	2.881
**5**	*5. Region*	18.683	2.220
**6**	*6. Region*	12.644	2.582
**7**	*7. Region*	12.602	2.951
**8**	*8. Region*	10.497	2.108
	**Total:**	**170.758**	**19.618**

Source: 1. https://www.endeksa.com/tr/analiz/turkiye/erzurum/demografi [Internet].
