# Supplementary material for: A case study of transferring the effect of demographic factors on e-waste recycling to the waste container assignment model
Source: PLoS One. 2025 Aug 25;20(8):e0315695. doi: 10.1371/journal.pone.0315695 (PMC12377600; doi:10.1371/journal.pone.0315695)
Supplement: S5 Table — (PDF) [file pone.0315695.s005.pdf]

S5 Table. Scoring for the education level

Education Level	***Education Score***
Primary school	1
High school	6
University	10
