# Supplementary material for: A case study of transferring the effect of demographic factors on e-waste recycling to the waste container assignment model
Source: PLoS One. 2025 Aug 25;20(8):e0315695. doi: 10.1371/journal.pone.0315695 (PMC12377600; doi:10.1371/journal.pone.0315695)
Supplement: S6 Table — (PDF) [file pone.0315695.s006.pdf]

S6 Table. Education level and age distribution by neighborhood

No	*Neighborhood Name*	*Educational Status*	*Scores for Education*	*Age Distribution*	*Scores for Age*
**1**	1. Region	*Primary school*	1	*0-19*	7
**2**	2. Region	*Primary school*	1	*0-19*	7
**3**	3. Region	*High school*	6	*20-29*	10
**4**	4. Region	*High school*	6	*20-29*	10
**5**	5. Region	*High school*	6	*20-29*	10
**6**	6. Region	*High school*	6	*20-29*	10
**7**	7. Region	*University*	10	*20-29*	10
**8**	8. Region	*High school*	6	*0-19*	7
	**Total**		**42**		**71**
