# Supplementary material for: A case study of transferring the effect of demographic factors on e-waste recycling to the waste container assignment model
Source: PLoS One. 2025 Aug 25;20(8):e0315695. doi: 10.1371/journal.pone.0315695 (PMC12377600; doi:10.1371/journal.pone.0315695)
Supplement: S7 Table — (PDF) [file pone.0315695.s007.pdf]

S7 Table. Population, income, education, and age ratios of neighborhoods

No	***Neighborhood***	***Population***	***Income Level***	***Education Level***	***Age Distribution***
**1**	*1. Region*	0.216	0.114	0.024	0.1
**2**	*2. Region*	0.189	0.105	0.024	0.1
**3**	*3. Region*	0.143	0.131	0.143	0.14
**4**	*4. Region*	0.134	0.147	0.143	0.14
**5**	*5. Region*	0.109	0.113	0.143	0.14
**6**	*6. Region*	0.074	0.132	0.143	0.14
**7**	*7. Region*	0.074	0.15	0.238	0.14
**8**	*8. Region*	0.061	0.107	0.143	0.1
