# Supplementary material for: A case study of transferring the effect of demographic factors on e-waste recycling to the waste container assignment model
Source: PLoS One. 2025 Aug 25;20(8):e0315695. doi: 10.1371/journal.pone.0315695 (PMC12377600; doi:10.1371/journal.pone.0315695)
Supplement: S8 Table — (PDF) [file pone.0315695.s008.pdf]

S8 Table. Ranking of regions according to harmonic mean values

No	***Neighborhood***	***Harmonic Mean Values***
**1**	*4. Region*	0.141
**2**	*3. Region*	0.139
**3**	*7. Region*	0.127
**4**	*5. Region*	0.125
**5**	*6. Region*	0.114
**6**	*8. Region*	0.094
**7**	*1. Region*	0.061
**8**	*2. Region*	0.060
