# Supplementary material for: A case study of transferring the effect of demographic factors on e-waste recycling to the waste container assignment model
Source: PLoS One. 2025 Aug 25;20(8):e0315695. doi: 10.1371/journal.pone.0315695 (PMC12377600; doi:10.1371/journal.pone.0315695)
Supplement: Attachment — Submitted filename: Manuscript-review.docx [file pone.0315695.s009.docx]

**A case study of transferring the effect of demographic factors on e-waste recycling to the waste container assignment model**

**Tuba BAZANCİR ^a^, Yeşim OK ^b*^**

**a** Trabzon University, Quality Coordination Department, Trabzon, TURKEY

**b** Ataturk University, Engineering Faculty, Industrial Engineering Department, Erzurum, TURKEY

*** Corresponding author**

**E-mail:** [yesim.ok@atauni.edu.tr](mailto:yesim.ok@atauni.edu.tr) **(YO)**

**Abstract**

Collecting waste directly from the consumer could be considered the initial stage of electronic waste recycling. Despite numerous studies employing mathematical assignment models to determine the optimal location of containers for waste collection from end-users, no research has been deemed to incorporate the demographic characteristics of the regions into these assignment models. This study aims to accomplish more effective results by incorporating the factors affecting consumers' recycling trends (the population, income level, education level, and age distribution of the relevant region) into the assignment model. By integrating abovementioned parameters characterizing regional differences in the micro sense and country-based differences in the macro sense into the assignment model, a generic model is structured to help local administrators determine their first-step e-waste recycling policies. A case study was carried out for a local government in Turkey which demonstrated that considerably more realistic results regarding the amount of e-waste to be collected can be obtained when factors indicating regional variances are included in the container assignment model. Local governments could utilize these findings as a reference to create more feasible policies for the following steps of e-waste recycling.

**Keywords**

*Container assignment; mathematical modeling; recycling affecting factors; municipal e-waste management*

1. **Introduction**

Electronic waste (e-waste) is one of the world's fastest-growing sources of waste (1,2). While the variety and quantity of electronic items grow in tandem with rapid changes in consumer behavior, their usable life reduces with every passing day. According to the Global E-waste Monitor Report 2020, 53.6 million tons of electronic waste were generated in 2019. It is anticipated that worldwide electronic waste will exceed 74 million tons by 2030. This number is nearly double the amount of e-waste generated in 2014. Furthermore, only 17.4% of the e-waste generated in 2019 was recycled officially (3).

The difficulty of managing waste electrical and electronic equipment has started to damage electronics' position as a "clean" technology, and the issue has grown in relevance as the electronics industry expands at one of the most rapid rates among all economic sectors (4).

E-waste left to nature could take several years to disintegrate, and the hazardous substances in its contents interacting with soil and water can cause a variety of detrimental effects. On the other hand, appropriately processed waste can provide a secondary source of raw materials for industry. Therefore, efficient management of electronic waste is vital for sustainability in many aspects. Even in developed countries, this recycling issue has not been completely and successfully addressed. For emerging countries like Turkey, it is a complex equation with many unknowns that require solutions. While it is acknowledged that manufacturers of electronic waste are involved in the process of implementing laws in developed economies, policymakers in developing countries frequently overlook the root causes because they place too much emphasis on the financial aspect of e-waste (5).

One of these sub-problems is allocating e-waste containers to places that will permit access to the most e-waste. Local governments often do not regard waste container assignment to be a serious issue, and therefore randomly allocate the same number of waste bins to all regions within their jurisdiction. As a result, they incur additional expenditures and produce inefficient results by failing to differentiate between locations with low and high e-waste generation potential. However, the question that must be addressed is: why does the amount of waste differ by region? Several crucial factors influence waste generation and container placement. Among these factors, it is obvious that the income and educational levels of electronic consumers, as well as the age and population size distribution in the surrounding area, are all significant.

The decision-makers in municipal solid waste management systems have to consider a number of processes, including waste collection, transportation, treatment, and disposal. Uncertainties also surround a large number of system characteristics, influencing factors, and their interconnections. These uncertainties may be further increased by temporal and spatial variations in numerous system components (6).

The primary goal of this study is to include crucial sociodemographic characteristics that influence e-waste generation and container placement into a mathematical model. Thus, local governments that use this generic model will be able to develop a specific application by incorporating their conditions into the model, meeting municipalities' demand for methodical and scientific studies to guide the e-waste collection process.

2. **Related Literature**

Various deterministic models have been used for different steps of e-waste recycling in the current literature. (7) proposed a reverse logistics network model that aims to reduce total cost taking into consideration the cost of collection, the cost of installing separation, repair, and recycling facilities, processing capacity, and transportation between e-waste facilities. For a more realistic LP model under uncertainty, (8) defined parameters such as demand (amount of e-waste to be processed), the operating capacity of resources, the quantity of output from e-waste, and processing times. (9) developed a binary mathematical model for integrated e-waste management at the national level for Greece and concluded that the most effective e-waste management strategy involves both mechanical recycling and e-waste export. It is claimed that for the first stage of e-waste management, 90% of recycling costs are labor costs in developed countries by (10).

Research on e-waste recycling has typically employed multi-purpose models to address both environmental and economic concerns (11). Among them, (2), proposed a DEMATEL method that reduces not only transportation costs but also transportation-related emissions and fossil fuel consumption, and (12) ) used the fuzzy DEMATEL approach to design an e-waste collection policy in India, taking into consideration a set of critical success factors such as technology participation, environmental programs, and so on.

Another issue that is under consideration is vehicle routing for e-waste pickup. In a case study, in southern Poland, (13) proposed a genetic algorithm-based model for planning the most appropriate daily assignment and routes for vehicles that demonstrated the effectiveness and optimizer-ability of mobile electronic waste collection. (14) proposed two models, flow-based and node-based, for the capacity location routing problem on behalf of a company dealing with financial incentive collection of used products from customers. (15) developed a mathematical model to identify the best locations for collecting e-waste and the best routes for vehicles based on opportunity costs.

Research that considers the influence of consumer attitudes on e-waste recycling has additionally received attention. Consumer participation has been considered one of the most critical factors contributing to the effectiveness of e-waste recycling management (16). Therefore, it is crucial to understand consumers' disposal and recycling behavior to increase residential recycling rates. Survey-based research makes up the majority of the literature on consumers' e-waste recycling behavior (17). Since the usage of electronic devices is most commonly associated with young people, surveys of university students come to the forefront (18,19). These surveys also have made it simpler to acquire data on the lifespan of electronic equipment (20,21).

Demographic profile data, such as gender, age, educational status, and monthly income, as well as subjective factors, such as awareness, responsibility, perceived benefits, and risks, are used as input data to determine the factors that might be useful in determining consumers' intentions of recycling e-waste in (22). Consequently, it was concluded that factors such as awareness, educational status, responsibility attribution, and age are among the important factors affecting consumers' e-waste recycling intentions, and rational factors outweigh normative factors.

To the best of the authors’ knowledge, this is the first attempt to incorporate demographic factors affecting the consumer's e-waste recycling intentions into the e-waste container assignment model in this study.

3. **Material and Methods**
   3.1. Classification of Electronic Waste

Electronic items turn into electronic waste when they complete their useful lives. Electronic waste is formed as follows: first, electronic goods are sold. Then these items are used in homes and workplaces. When they complete their useful lives, electronic waste is formed. Finally, they take part in the collection and recycling phases. E-waste encompasses a wide range of products.

The European Union (EU) Waste Electrical and Electronic Equipment (WEEE) Directive divides e-waste into six categories (as shown in Figure 1) based on recycling and recovery targets (23).

**Figure 1.** WEEE Categories

While the study's default e-waste collection containers are suitable for categories 2 and 6 (colored red in Figure 1), the suggested model's general form makes it possible to apply to additional categories.

The majority of research in the e-waste literature used the amount of e-waste as a parameter in their models. In contrast, in this study, it is derived from the model solution. The main purpose of this study is to create a generic assignment model based on the factors affecting electronic waste generation.

3.2. Factors Affecting E-waste Recycling

When some of the studies are examined regarding the willingness for e-waste recycling; providing ease of collection and receiving some financial incentives for product returns work well (24), gender, age, and environmental awareness are important determinants (25), as are age, general awareness and general attitude towards the environment (26).

According to a survey in the USA (27), the strongest determinants of the intention to recycle e-waste are personal and moral norms, environmental awareness, and social expectations, while gender, marital status, and suitability of recycling have a smaller effect. Through hypothesis testing using survey data, a WEEE recycling behavior model for Romanians was developed, and it was found that people's attitudes and habits were the main determinants of their e-waste recycling behavior (28). Using the logistic regression model, it is also found that education level, age, and household income are important and dominant factors in WEEE behavior (29).

**Figure 2.** Country-based differences affecting WEEE consumption in the macro sense and Socio-economic/demographic factors affecting WEEE behavior in the micro sense

Studies conducted in different countries have shown that various determinants, such as socio-economic and demographic factors, environmental awareness, habits, convenience, etc., can be used to analyze e-waste recycling behaviors. There are some contradictions in the results, indicating that the unique characteristics of each country have different effects on people's participation in e-waste collection and recycling activities. As an addition to the literature, this study is the first to include the factors that affect e-waste demand in the assignment model, in contrast to other studies that assign containers solely based on waste demand.

3.3. Model Assumptions

Various scenarios concerning the collection and transportation of electronic waste may require optimization. The location of collection sites and treatment facilities is one of the most frequently addressed subjects among them. One aspect that influences consumers' propensity to recycle is the collection point's proximity to demand (14). Unlike earlier research (30,31), the model proposed in this study incorporates the factors affecting electronic waste recycling into the assignment model as well.

The assumptions of this study could be listed as follows:

- The model incorporates a capacity restriction to represent its assumption that every neighborhood generates e-waste demand based on its unique socio-demographic characteristics.
- Due to the abovementioned factors being incorporated as parameters in the container assignment model, the e-waste amount is predictable approximately.
- As the harmonic mean eliminates the extreme points and reduces the effect of averaging the high value between the data, it is preferred in this study for factors combination.
- Even though the application is intended for a local government region in Turkey, as the model has a generic structure because of its general parameters, local policymakers in any nation could use it to gain insight while determining their initial e-waste policies.
  3.4. Model Formulation

We define the identity set of all demand nodes (DN) as I= {i| i=1, 2, 3… m} and the identity set of all candidate container points (CP) as J= {j| j=1, 2, 3…n}.

*DC* is the maximum coverage distance between each DN and its corresponding candidate CP. Therefore, for each DN, the identity set of candidate CPs to which a DN can be assigned is calculated as *𝑁_𝑖_* = [j | d_ij_ ≤ *Dc*, Ɐj ϵ J] Ɐi ϵ I, where d_ij_ is the distance between i^th^ DN to the j^th^ CP.

Also, the following parameters are defined for model formulation:

CN (i) is the number of containers in ith DN, where CNmax is the maximum number of containers that can be placed with the budget available. Here, the budget constraint allocated by municipalities for e-waste recycling is considered.

Since e-waste recycling rates vary from country to country, two more parameters were added to the model formulation. Ewcr is the e-waste collection percentage by country and Ewcp is the e-waste amount per household by country (per house).

Except for these parameters, HN (i) is the number of houses in ith DN and S is the capacity of each container, as the parameter in which socio-demographic factors of the relevant neighborhood are included in the model. HMF (i) is the district-based harmonic mean of socio-demographic factors affecting e-waste recycling.

The decision variables in the model are:

The objective function is:

(1)

The constraints are:

(2)

(3)

(4)

(5)

 (6)

$HMF(i)=\frac{4}{\text{(}\frac{1}{\text{t}_{1} }\text{+}\frac{1}{\text{t}_{2} }\text{+}\frac{1}{\text{t}_{3} }\text{+}\frac{1}{\text{t}_{4} }\text{)}} , \forall i \in I$ (7)

$x_{ij} \in\left\{0,1\right\} and y_{ij} \geq0 \forall i \in I, \forall j\in J$ (8)

Equation 1 aims to maximize the amount of e-waste to be collected. Equation 2 ensures that e-waste containers are assigned to every neighborhood within the coverage area. This uses the previously described formulation of the set Ni. Candidate container allocations are limited to regions within the maximum coverage distance, similar to the model used by (32) for the distributed energy network design. Equation 3 limits the number of containers for each region according to the general situation. Equation 4 is a budget constraint. The maximum number of containers that can be used in the model is obtained by dividing the budget of the relevant local administration by the container unit cost. Equation 5 limits the maximum quantity of e-waste for a region. Since the model is generic, this value can be calculated by multiplying the number of houses in the relevant region by the amount of e-waste per household by country and the e-waste collection percentage for each country according to the global e-waste statistics. Equation 6 is the capacity constraint and the harmonic mean value of factors (HMF) in Equation 6 is derived from Equation 7. The harmonic mean values for each neighborhood are multiplied by container capacity, resulting in an impact restricting the amount of e-waste in the model. Equation 7 includes the harmonic mean formula. The values t_1_, t_2_, t_3_, and t_4_ reflect the population, income, education, and age distribution ratios, respectively. Finally, Equation 8 defines the decision variable x as 0-1, and y as a continuous variable.

The model comes to the fore with a different perspective proposal as it creates an e-waste demand based on the different characteristics of each neighborhood. The harmonic mean is preferred in this model because it eliminates the extreme points and reduces the effect of averaging the high value between the data.

4. **Case Study**

**Figure 3.** The general flow of the proposed approach

4.1. The general flow of the proposed approach

Figure 3 demonstrates the flow of the novel approach that has been suggested, in which the parameters characterizing local differences in the micro sense and country-based differences in the macro sense are both integrated into the assignment model.

4.2. Constitution of Parameters
     4.2.1. *Number of houses in selected Neighborhood (HN (i))*

The number of houses belonging to the selected Neighborhood has been given (in Table 1, in the appendix).

4.2.2. *Distances between demand points and candidate container points (d_ij_).*

Euclidean distances are calculated using random coordinates generated at specific intervals for the demand and candidate container points that present the Euclidean distances, 𝑑_𝑖𝑗_, between demand point 𝑖 and candidate container 𝑗 in meter scale (Table 2, in the appendix).

4.2.3. *E-Waste Amount per Household	(Ewcp)*

According to the data in the Beyond Waste report, the total amount of e-waste in Turkey for 2016 is 542 thousand tons, while the amount of e-waste in the televisions /monitors and informatics-telecommunication equipment categories of e-waste categories is 117 thousand tons (33)*.*

As mentioned before, e-waste is classified into six categories. Since the televisions /monitors and informatics-telecommunication equipment categories of e-waste are taken into consideration in this study, an approximate percentage is obtained by dividing the e-waste amount in these two categories by the total e-waste amount (Equation 9).

$117/542 =0,215$ (9)

According to the research conducted by the “Clear it waste collection” company; e-waste amounts per household have been determined for some European countries (34) It has been determined that the amount of e-waste per household in Turkey is 41.8 kg (“Research: Electronic waste per household in Turkey was 41.8 kilograms - Last Minute World News | NTV News,’” 2021). The amount of e-waste categories of televisions /monitors and informatics-telecommunication equipment per household is calculated as in Equation 10. Salihoğlu and Kahraman's study (36) evaluated the e-waste consumption in Bursa, Turkey through a survey, and they concluded that the annual consumption amount was 8.14 kg/person. This outcome coincides with the proportionate calculation derived by equation 10.

$41,8*0,215 = 8,987= 9 kg/year$ (10)

So, the e-waste amount per household by country *(Ewcp)* is calculated for Turkey as 9 kilograms. The monthly value is calculated by dividing the annual income per household by 12 in Turkey, as in equation 11.

$9/12=0,75 kg/month$ (11)

Here, the 0.75 value obtained for e-waste categories of televisions /monitors and informatics-telecommunication equipment, will be multiplied by the number of houses in each neighborhood, in the constraint in Equation 5 in the model.

4.2.4. *E-waste collection rate by country (Ewcr)*

The e-waste collection rate varies country by country. The E-waste collection percentage for Turkey (E_wcr_) was 0.18 in 2019 (37)*.*

4.2.5. *Capacity of an e-waste container (S)*

S is the container capacity; the capacity of a container is taken as 1500 kg. A larger capacity is not needed as it is for only televisions /monitors and informatics-telecommunication equipment.

4.2.6. *Maximum number of containers (CN_max_)*

The budget allocated by the municipality for e-waste containers is taken as 300.000 ₺. The cost of the unit e-waste container is determined as 13.000 ₺ in line with the information obtained from authorized institutions. The municipality's budget is divided by the unit box cost and the maximum number of containers that can be used in the selected neighborhoods is calculated as in Equation 12.

CN _max_ = Total budget of Municipality / Unit box cost

$300.000/13.000=23$ (12)

4.2.7. *Candidate Container numbers for each neighborhood CN (i)*

The maximum number of containers is calculated above, taking into account the budget allocated to e-waste recycling by the local government. An approximate value that will not fall below this CN max value can be taken as the total number of containers. Thus, alternative candidate appointment points would be obtained. It is determined above that 23 containers would be placed in total due to the budget constraint. The distribution of container candidate points has been determined (in Table 3, in the appendix).

**Figure 4.** Candidate points for e-waste containers in Google Earth

The placement of potential locations for e-waste containers in the neighborhoods in the Google Earth software is shown in Figure 4. When placing candidate points on the map, neighborhood boundaries are considered, and allocations have been made randomly.

4.2.8. *Calculation of Harmonic Mean Values*

The Index Platform obtains per capita income and population information for all neighborhoods (38). (in Table 4, in the appendix).

It has been observed that the use of electronic goods is associated with the level of education. According to the results of (20); it is concluded that the age range of 18-24 is the age range in which most electronic devices are used, therefore the potential to create televisions /monitors and informatics-telecommunication equipment categories of e-waste is much higher in the young age group than the middle and advanced. In this context, in our study, a scale between 1 and 10 is used for scoring the level of education.

Neighborhoods where the 20-29 age range is predominant are given 10 points, and 7 points for the 0-19 age range. The education level scores, education scoring, and age distribution scoring have been determined (in Table 5 and Table 6, in the appendix).

The population ratio for all neighborhoods is normalized by dividing the population of each neighborhood by the total population. The same normalization process is performed for per capita income, education, and age distribution scores, and the relevant ratios are calculated (in Table 7, in the appendix).

After the rate calculation of the four factors for all neighborhoods, these ratios have been reversed in turn, calculating the equal weight harmonic means for each one. For example, for the 1^st^ Region (Shukrupasa Neighborhood), the equally weighted harmonic mean value is calculated as in Equation 13.

$HMF(1)=\frac{4}{\left(\frac{1}{0,216}+\frac{1}{0,114}+\frac{1}{0,024}+\frac{1}{0,10}\right)}=0,061$ (13)

According to the calculated harmonic means, the neighborhoods have been sorted (in Table 8 in the appendix), starting with the larger one.

The harmonic mean values of the four factors that are frequently underlined in the literature regarding e-waste consumption are calculated with the formula in Equation 7 and used in the mathematical model. The reason for choosing the harmonic mean is to eliminate the extreme values.

5. **Results**
   5.1. Scenario Analysis

The results of the proposed model were evaluated with different scenario analyses.

**Table 1.** Objective function values for models include and exclude factors in different coverage distances.

|  | | *Objective Function Value*  *(Amount of e-waste collected) (kg)* | | | | | |
| --- | --- | --- | --- | --- | --- | --- | --- |
| *Coverage Distance - Dc (m)* | **500** | | **750** | **1000** | **1500** | **2000** | **2500** |
| *Factors Included* | 1396.5 | | 2463 | 2956.5 | 3570 | 3972 | 3972 |
| *Factors Not Included* | 13500 | | 22500 | 27000 | 31500 | 34500 | 34500 |

In this context, the effect of adding macro and micro parameters to the assignment model for e-waste container assignment on the result obtained is given in Tables 1 and 2 at different coverage distance sizes. When the results in both tables are examined, the results can be evaluated in two dimensions.

**Table 2.** Results for models including and excluding factors in different coverage distances.

| ***Number of containers located*** | | | | | | | ***Number of assignments*** | | | | | |
| --- | --- | --- | --- | --- | --- | --- | --- | --- | --- | --- | --- | --- |
| ***Coverage Distance - Dc (m)*** | **500** | **750** | **1000** | **1500** | **2000** | **2500** | **500** | **750** | **1000** | **1500** | **2000** | **2500** |
| ***Factors Included*** | 10 | 16 | 16 | 15 | 15 | 15 | 13 | 17 | 18 | 21 | 23 | 23 |
| ***Factors Not Included*** | 10 | 16 | 16 | 13 | 14 | 16 | 13 | 17 | 18 | 21 | 23 | 23 |

Initially, the parameters added to the proposed model have a greater impact on the objective function: the amount of e-waste expected to be collected so much so that the expected amount of waste could be almost ten times higher when the factors are excluded.

Secondly, as the coverage distance increases, the number of possible containers (that is, the number of assignments) in which each neighborhood can throw its waste increases, but it is seen that the number of containers assigned decreases relatively. When the coverage distance is changed on the scale of 500–2500 meters, the number of containers placed by the model varies between 10 and 16. The number of zones assigned to containers increases as the coverage distance increases. What is meant by increasing the coverage distance here is to take into account the possibility of consumers living in a neighborhood throwing their waste into containers further away from them. However, in actuality, it is more likely that as it becomes more difficult for end users to access e-waste recycling containers, their willingness to recycle decreases.

By extending the scenario analysis, the container assignment results of the parameters added to the model for Dc = 1000 m and Dc = 2000 m are given in detail in Tables 3 (a) and (b).

**Table 3.** (a) Assignment results for maximum coverage distance of 1000 m (b) Assignment results for maximum coverage distance of 2000 m

| (a) Dc=1000 m | | | | | | | | | (b) Dc= 2000 m | | | | | | | | | |
| --- | --- | --- | --- | --- | --- | --- | --- | --- | --- | --- | --- | --- | --- | --- | --- | --- | --- | --- |
|  | ***Regions*** | | | | | | | |  | ***Regions*** | | | | | | | | |
| **Z=2956.5** |  |  |  |  |  |  |  |  | **Z=3972** |  |  |  |  |  |  |  |  |  |
| **Candidate Points** | **1** | **2** | **3** | **4** | **5** | **6** | **7** | **8** | ***Candidate Points*** | **1** | **2** | **3** | **4** | **5** | **6** | **7** | **8** |  |
| 1 | 1 | - | - | - | - | - | - | - | *1* | 1 | - | - | - | - | - | - | - |  |
| 2 | - | - | - | - | - | - | - | - | *2* | - | 1 | - | - | 1 | - | - | - |  |
| 3 | - | - | - | - | - | - | - | - | *3* | 1 | - |  | - | - | - | - | - |  |
| 4 | - | - | - | - | 1 | - | - | - | *4* | 1 | 1 | - | 1 | 1 | 1 | - | - |  |
| 5 | - | 1 | - | - | - | - | - | - | *5* | - | - | - | - | - | - | - | - |  |
| 6 | - | 1 | - | - | - | - | - | - | *6* | - | - | - | - | - | - | - | - |  |
| 7 | - | 1 | - | - | - | - | - | - | *7* | - | - | - | - | - | 1 | - | - |  |
| 8 | - | 1 | - | - | - | - | - | - | *8* | - | - | - | - | - | - | 1 | - |  |
| 9 | - | - | 1 | - | - | - | - | - | *9* | - | - | 1 | - | - | - | - | - |  |
| 10 | - | - | - | - | - | - | - | - | *10* | 1 | - | 1 | - | - | - | - | - |  |
| 11 | - | - | - | - | - | - | - | - | *11* | - | - | 1 | - | 1 | - | - | - |  |
| 12 | - | - | - | - | 1 | - | - | - | *12* | - | - | - | 1 | - | - | - | - |  |
| 13 | - | - | - | 1 | - | - | - | - | *13* | - | - | - | 1 | - | - | - | - |  |
| 14 | - | - | - | 1 | - | 1 | - | - | *14* | - | - | - | - | - | - | - | - |  |
| 15 | - | - | - | - | - | - | - | - | *15* | - | 1 | - | - | - | - |  | - |  |
| 16 | - | - | - | 1 | - | - | - | - | *16* | - | - | - | - | - | - | - | - |  |
| 17 | - | - | - | - | - | - | - | - | *17* | - | - | - | - | - | - | - | - |  |
| 18 | - | - | - | - | - | - | - | - | *18* | - | - | - | - | - | - | 1 | 1 |  |
| 19 | - | - | - | - | - | 1 | - | - | *19* | - | 1 | - | - | - | - | - | - |  |
| 20 | - | - | - | - | - | - | 1 | - | *20* | - | - | - | - | - | - | - | 1 |  |
| 21 | - | - | - | - | 1 | - | - | - | *21* | - | - | - | - | - | - | - | - |  |
| 22 | - | - | - | - | - | - | 1 | 1 | *22* | - | - | - | - | - | - | - | - |  |
| 23 | - | - | - | - | - | - | - | 1 | *23* | - | - | - | - | - | - | - | - |  |
| **CN** | **1** | **4** | **1** | **3** | **3** | **2** | **2** | **2** | ***CN*** | **4** | **4** | **3** | **3** | **3** | **2** | **2** | **2** |  |

The objective function value of the model with 1000 m Dc value (2956.5 kg) is lower than the objective function value of the model with 2000 m Dc (3972 kg). Therefore, when the maximum coverage distance is reduced, it is seen that the number of containers increases, but the expected amount of e-waste decreases, too.

The assignments changing depending on the maximum coverage distance determined in the model are visualized in Figures 5 and 6. Comparing above mentioned figures, it is obvious that as the coverage distance between the end user and the e-waste containers increases, fewer containers are allocated (16 containers in Figure 5 and 14 containers in Figure 6).

**Figure 5.** Assignment results for maximum coverage distance of 1000 m (16 containers)

This is because, in regions where neighborhood boundaries are close together, multiple neighborhoods can use the same container as is already the practice case. Figure 6 illustrates this scenario with the assignment of three different regions (4, 5, and 8) to container number 18.

**Figure 6.** Assignment results for maximum coverage distance of 2000 m (15 containers)

Because of this, the coverage distance should be determined by considering the necessity of not taking the maximum coverage distance too high and placing e-waste recycling bins at points where the end user can reach them as easily as possible. In other words, the coverage distance should not be chosen as a bigger or a smaller value instead of this, the most appropriate distance should be determined with various trials.

6. **Discussion and Conclusions**

E-waste recycling is crucial to sustainability as it reduces waste, minimizes damage to the environment, conserves resources, and maintains a circular approach to electronics production and disposal.

Essentially, the main motivation of this study is to create a general framework that will aid in the development of local e-waste collection policies based on the main factors that cause regional variations in the recycling rate of e-waste. Even though numerous research have discussed these factors none of them directly have included these in the waste container assignment model. The regional ratios derived from these factors have been integrated into the e-waste consumption capacity constraint through a harmonic mean.

The suggested approach varies from the conventional cluster coverage model since this model's objective function is to maximize the amount of e-waste. This use of maximum coverage distance is similar to the distributed energy network design study by Ok and Atak (32).

In summary, the main motivation of this proposed approach is to incorporate the macro and micro factors that can determine the e-waste production potential into the model, which varies in regions with high and low e-waste production potential.

Although the model is mainly focused on waste container assignment, it appears from the results that the factors included in the proposed model do not have much impact on the number of containers placed. This is an expected result, since the constraint of assigning at least one container to each region is already included in the model. It seems that the difference in the number of containers is mostly due to the change in the coverage distance included in the model.

However, the main contribution of the model proposed here is in determining the amount of waste likely to be collected. As a result of the proposed model, regional variations come to the fore, thus paving the way for more realistic waste collection values to be obtained.

As mentioned before, e-waste collection management consists of many sequential steps, and each step has an impact on the next. The more accurately the amount of waste to be collected is estimated in the waste container placement process, which is considered as the first step, the more accurately and effectively the policies regarding the next step, collection and separation/disposal processes, can be determined.

It should also be noted that the fact that the results obtained have not been proven to be accurate in a joint project with the municipality where the application was implemented can be considered as the weak point of the study.

As a recommendation for future research, it might be evaluated what kind of results would be obtained when the e-waste factors are included in the model with different weights, and different criteria could be used. Additionally, costs could be added to the model as a constraint, and the proposed approach could be used for different factors affecting recycling and for different categories of WEEE for different countries. Moreover, to evaluate and measure the success of recycling activities, the recycling rate, which calculates the percentage of waste, could be considered if the implementation of the proposed model could be carried out in a joint project with the local government.

**Statements and Declarations**

**Competing Interests**

The authors have no competing interests to declare that are relevant to the content of this article.

**Funding**

The authors did not receive support from any organization for the submitted work.

**Authors’ Contributions**

***YO:*** Conceptualization; Formal analysis; Methodology; Software; Validation, Visualization, Supervision; Writing - original draft; and review & editing.

***TB****:* Investigation, Conceptualization; Visualization.

**References**

1.	Boubellouta B, Kusch-Brandt S. Relationship between economic growth and mismanaged e-waste: Panel data evidence from 27 EU countries analyzed under the Kuznets curve hypothesis. Waste Manag [Internet]. 2021 [cited 2022 Nov 3];(120):85–97. Available from: https://www.sciencedirect.com/science/article/pii/S0956053X20306486

2.	Liang S, Pi H, Hsu C, Kadry S. Sustainable transportation system for electronic waste recycling from a social perspective. IET Intell Transp Syst [Internet]. 2020 Nov 1 [cited 2024 Apr 17];14(11):1378–87. Available from: https://ietresearch.onlinelibrary.wiley.com/doi/abs/10.1049/iet-its.2019.0824

3.	Vanessa F, Baldé CP, Ruediger Kuehr GB. E-Waste Statıstıcs Guıdelınes On Classıfıcatıon, Reportıng And Indıcators E-Waste Statıstıcs Guıdelınes On Classıfıcatıon, Reportıng And Indıcators Second Edıtıon. 2020 [cited 2024 Apr 17]; Available from: http://ewastemonitor.info/

4.	Hischier R, Achachlouei MA, Hilty LM. Evaluating the sustainability of electronic media: Strategies for life cycle inventory data collection and their implications for LCA results. Environ Model Softw. 2014 Jun 1;56:27–36.

5.	Méndez-Fajardo S, Böni H, Vanegas P. Improving sustainability of E-waste management through the systemic design of solutions: the cases of Colombia and Ecuador. Handb Electron Waste Manag [Internet]. 2020 [cited 2022 Nov 1]; Available from: https://www.sciencedirect.com/science/article/pii/B9780128170304000127

6.	Guo P, Huang GH, He L, Sun BW. ITSSIP: Interval-parameter two-stage stochastic semi-infinite programming for environmental management under uncertainty. Environ Model Softw. 2008 Dec 1;23(12):1422–37.

7.	Elbadrawy R, Moneim A, Fors M. E-waste reverse logistic optimization in Egypt. IEOM 2015 - 5th Int Conf Ind Eng Oper Manag Proceeding. 2015 Apr 23;

8.	Polat O, Capraz O, Gungor A. Modelling of WEEE recycling operation planning under uncertainty. J Clean Prod. 2018 Apr 10;180:769–79.

9.	Aidonis D, Achillas C, Folinas D, Keramydas C, Tsolakis N. Decision Support Model for Evaluating Alternative Waste Electrical and Electronic Equipment Management Schemes—A Case Study. Sustainability [Internet]. 2019 Jun 18 [cited 2023 Aug 17];11(12):3364. Available from: https://www.mdpi.com/2071-1050/11/12/3364/htm

10.	Dias P, Bernardes A, Huda N. Ensuring best E-waste recycling practices in developed countries: An Australian example. J Clean Prod. 2019 Feb 1;209:846–54.

11.	Šomplák R, Kůdela J, Smejkalová V, Nevrlý V, Pavlas M, Hrabec D. Pricing and advertising strategies in conceptual waste management planning. J Clean Prod. 2019 Dec 1;239:118068.

12.	Singh S, Dasgupta MS, Routroy S. Analysis of Critical Success Factors to Design E-waste Collection Policy in India: A Fuzzy DEMATEL Approach. Environ Sci Pollut Res. 2022 Feb 1;29(7):10585–604.

13.	Nowakowski P, Król A, Mrówczyńska B. Supporting mobile WEEE collection on demand: A method for multi-criteria vehicle routing, loading and cost optimisation. Waste Manag. 2017 Nov 1;69:377–92.

14.	Hosseini M, Dehghanian F, Salari M. Selective capacitated location-routing problem with incentive-dependent returns in designing used products collection network. Eur J Oper Res. 2019 Jan 16;272(2):655–73.

15.	Aquino R, Ferreira da Silva Junior I, Guarnieri P, Camara e Silva L. The proposition of a mathematical model for the location of electrical and electronic waste collection points. Sustainability [Internet]. 2020 [cited 2023 Jan 5];13(1)(224). Available from: https://www.mdpi.com/939388

16.	Borthakur A, Govind M. Emerging trends in consumers’ E-waste disposal behaviour and awareness: A worldwide overview with special focus on India. Resour Conserv Recycl. 2017 Feb 1;117:102–13.

17.	Dhir A, Koshta N, Goyal R, LastName… MS. Behavioral reasoning theory (BRT) perspectives on E-waste recycling and management. J Clean Prod [Internet]. 2021 [cited 2022 Nov 3];(280). Available from: https://www.sciencedirect.com/science/article/pii/S0959652620343146

18.	Singhal D, Tripathy S, Jena SK. Sustainability through remanufacturing of e-waste: Examination of critical factors in the Indian context. Sustain Prod Consum [Internet]. 2019 [cited 2023 Aug 17];20:128–39. Available from: https://www.sciencedirect.com/science/article/pii/S2352550918304093

19.	Aboelmaged M. E-waste recycling behaviour: An integration of recycling habits into the theory of planned behaviour. J Clean Prod [Internet]. 2021 [cited 2022 Nov 3];(278). Available from: https://www.sciencedirect.com/science/article/pii/S095965262034227X

20.	Islam M, Dias P, Huda N. Young consumers’e-waste awareness, consumption, disposal, and recycling behavior: A case study of university students in Sydney, Australia. J Clean Prod [Internet]. 2021 [cited 2022 Nov 3];(282). Available from: https://www.sciencedirect.com/science/article/pii/S0959652620345340

21.	Kankanamge CE. Consumer Behavior in the Use and Disposal of Personal Electronics: a Case Study of University Students in Sri Lanka. Circ Econ Sustain. 2023 Mar;3(1):407–24.

22.	Nguyen H. Integrating the valence theory and the norm activation theory to understand consumers’e-waste recycling intention. Chinese J Popul Resour Environ [Internet]. 2023 [cited 2024 May 3]; Available from: https://www.sciencedirect.com/science/article/pii/S2325426223000037

23.	Directive EC. Official Journal of the European Union L. 2012 [cited 2024 May 10]. p. 38–71 Directive 2012/19/EU of the European Parliament and of the Council of 4 July 2012 on waste electrical and electronic equipment, WEEE. Available from: https://scholar.google.com/scholar?hl=tr&as_sdt=0%2C5&q=Directive+2012%2F19%2FEU+of+the+European+Parliament+and+of+the+Council+of+4+July+2012+on+waste+electrical+and+electronic+eq&btnG=

24.	Cooper T, Mayers K. Prospects for household appliances [Internet]. 2000 [cited 2023 Sep 7]. Available from: https://irep.ntu.ac.uk/id/eprint/6671/1/201121_7265 Cooper Publisher rescanned.pdf

25.	Saphores J. Household willingness to recycle electronic waste: an application to California. Environ Behav [Internet]. 2006 Mar [cited 2023 Sep 7];38(2):183–208. Available from: https://journals.sagepub.com/doi/abs/10.1177/0013916505279045

26.	Nnorom I, Ohakwe J, Osibanjo O. Survey of willingness of residents to participate in electronic waste recycling in Nigeria–A case study of mobile phone recycling. J Clean Prod [Internet]. 2009 [cited 2023 Sep 7]; Available from: https://www.sciencedirect.com/science/article/pii/S0959652609002534

27.	Saphores J, Ogunseitan O, Shapiro A. Willingness to engage in a pro-environmental behavior: An analysis of e-waste recycling based on a national survey of U.S. households. Resour Conserv Recycl. 2012 Mar 1;60:49–63.

28.	Colesca SE, Ciocoiu CN, Popescu ML. Determinants of WEEE recycling behaviour in Romania: a fuzzy approach. Int J Environ Res [Internet]. 2014 [cited 2023 Sep 1];8(2):353–66. Available from: https://www.sid.ir/en/VEWSSID/J_pdf/108220140213.pdf

29.	Song Q, Wang Z, Li J. Residents’ behaviors, attitudes, and willingness to pay for recycling e-waste in Macau. J Environ Manage [Internet]. 2012 [cited 2023 Sep 8]; Available from: https://www.sciencedirect.com/science/article/pii/S0301479712001557

30.	Nduneseokwu C, Qu Y, Appolloni A. Factors Influencing Consumers’ Intentions to Participate in a Formal E-Waste Collection System: A Case Study of Onitsha, Nigeria. Sustainability [Internet]. 2017 [cited 2023 Sep 1];9(6):881. Available from: https://www.mdpi.com/2071-1050/9/6/881

31.	Favot M, Grassetti L. E-waste collection in Italy: Results from an exploratory analysis. Waste Manag [Internet]. 2017 [cited 2023 Sep 1];(67):222–31. Available from: https://www.sciencedirect.com/science/article/pii/S0956053X17303495

32.	Ok Y, Atak M. Allocation of distributed energy systems at district-scale over wide areas for sustainable urban planning with a MILP model. Math Probl Eng [Internet]. 2018 [cited 2023 Sep 1]; Available from: https://www.hindawi.com/journals/mpe/2018/4208415/

33.	Akpulat O. Atigin Otesinde 2020.pdf vestel - Dünyada ve Türkiye’de Elektronik Atık Mevcut Durumu Araştırma Raporu [Internet]. 2020 [cited 2024 Apr 18]. Available from: https://www.google.com/search?q=atigin+Otesinde+2020.pdf+vestel&sca_esv=515e485ef43ad46e&ei=ky4hZoK6HLaQxc8P0Omc0Ao&udm=&ved=0ahUKEwjCz8aH_cuFAxU2SPEDHdA0B6oQ4dUDCBA&oq=atigin+Otesinde+2020.pdf+vestel&gs_lp=Egxnd3Mtd2l6LXNlcnAiH2F0aWdpbiBPdGVzaW5kZSAyMDIw

34.	https://www.clearitwaste.co.uk/ [Internet]. [cited 2024 Apr 18]. Available from: https://www.google.com/search?q=https%3A%2F%2Fwww.clearitwaste.co.uk%2F&sca_esv=515e485ef43ad46e&ei=UjAhZoWUK5fixc8PvaungA8&udm=&ved=0ahUKEwiFhejc_suFAxUXcfEDHb3VCfAQ4dUDCBA&uact=5&oq=https%3A%2F%2Fwww.clearitwaste.co.uk%2F&gs_lp=Egxnd3Mtd2l6LXNlcnAaAhgCI

35.	“Research: Electronic waste per household in Turkey was 41.8 kilograms - Last Minute World News | NTV News,.” 2021 [cited 2024 Apr 18]; Available from: https://www.ntv.com.tr/galeri/dunya/arastirma-turkiyede-hane-basi-elektronik-atik-41-8-kilogram-oldu,AkhYxuN-BE-Apz8dG8a0Fg

36.	Salihoğlu G, Kahraman A. Electric and electronic waste generation in Turkey: Bursa example. Uludağ Univ Eng Fac J [Internet]. 2016 [cited 2023 Aug 17];21. Available from: https://acikerisim.uludag.edu.tr/handle/11452/12159

37.	Global E-waste Monitor Report 2020 [Internet]. 2020 [cited 2024 Apr 17]. Available from: https://collections.unu.edu/eserv/UNU:6477/RZ_EWaste_Guidelines_LoRes.pdf

38.	https://www.endeksa.com/tr/analiz/turkiye/erzurum/demografi [Internet]. [cited 2024 Apr 18]. Available from: https://www.endeksa.com/tr/analiz/turkiye/erzurum/demografi
