# Supplementary material for: A case study of transferring the effect of demographic factors on e-waste recycling to the waste container assignment model
Source: PLoS One. 2025 Aug 25;20(8):e0315695. doi: 10.1371/journal.pone.0315695 (PMC12377600; doi:10.1371/journal.pone.0315695)
Supplement: Attachment — Submitted filename: Response to Reviewers.docx [file pone.0315695.s010.docx]

**Response to Editor**

**Comment 1:**

Please ensure that your manuscript meets PLOS ONE's style requirements, including those for file naming.

**Response 1:**

PLOS ONE's style requirements have been checked, and necessary adjustments have been made.

**Comment 2:**

Please ensure that you refer to Figure 2 in your text as, if accepted, production will need this reference to link the reader to the figure.

**Response 2:**

Figure 2 is linked to the body text of the manuscript previous paragraph on page 4.

**Comment 3:**

We note that Figures 4 in your submission contain [map/satellite] images which may be copyrighted.

We require you to either (1) present written permission from the copyright holder to publish these figures specifically under the CC BY 4.0 license, or (2) remove the figures from your submission.

**Response 3:**

The Google Earth screenshot in Figure 4 does not serve as a primary figure for the study; it has been included solely to offer a basic illustration. In this context, as you have indicated, *it was extracted from the main text* and *substituted with a visual representation illustrating* the relationship between candidate container points and demand points.

**Figure 4.** Candidate container and demand points relationship illustration

**Comment 4**

Please include captions for your Supporting Information files at the end of your manuscript, and update any in-text citations to match accordingly as stated in https://journals.plos.org/plosone/s/supporting-information

**Response 4**

We have included captions for *Supporting Tables* at the end of our manuscript, and updated all in-text citations to match accordingly.

**Comment 5:**

Please review your reference list to ensure that it is complete and correct. If you have cited papers that have been retracted, please include the rationale for doing so in the manuscript text, or remove these references and replace them with relevant current references. Any changes to the reference list should be mentioned in the rebuttal letter that accompanies your revised manuscript.

**Response 5:**

The reference list has been checked. No retracted article is found.

The following references have been added to the manuscript in response to referee comments:

1. Pakpour AH, Zeidi IM, Emamjomeh MM, Asefzadeh S, Pearson H. Household waste behaviours among a community sample in Iran: An application of the theory of planned behaviour. Waste Manag. 2014 Jun 1;34(6):980–6.
2. Sidique SF, Lupi F, Joshi S V. The effects of behavior and attitudes on drop-off recycling activities. Resour Conserv Recycl. 2010 Jan 1;54(3):163–70.
3. Xu L, Ling M, Lu Y, Shen M. Understanding Household Waste Separation Behaviour: Testing the Roles of Moral, Past Experience, and Perceived Policy Effectiveness within the Theory of Planned Behaviour. Sustainability. 2017 Apr 17;9(4):625.
4. Quynh Vo C, Samuelsen PJ, Leikny Sommerseth H, Wisløff T, Wilsgaard T, Elise Eggen A. Comparing the sociodemographic characteristics of participants and non-participants in the population-based Tromsø Study. BMC Public Health. 2023 Dec 1;23(1).

**Review Comments to the Author**

**Responses to Reviewers**

**Reviewer #1:**

**Comment 1:**

The abstract is comprehensive but could be more concise. Ensure it highlights key findings and the significance of the study more clearly.

**Response 1:**

***This paragraph is added to the Abstract.***

“The main contribution of the proposed model is that significantly more realistic results regarding the amount of e-waste to be collected can be obtained when factors indicating regional differences are included in the container assignment model. Thus, local governments could utilize these findings as a reference to create more sustainable policies for the next steps of e-waste recycling. The model application is demonstrated through a case study for a local government in Turkey.”

**Comment 2:**

Enhance the discussion on the gap in the literature that this study aims to fill.

**Response 2:**

***This paragraph is added to the last paragraph of Literature:***

Previous studies examining the factors influencing e-waste recycling behavior are predominantly survey-based, aimed at delineating the consumer profile. In summary, despite numerous studies addressing the determinant factors, none have concentrated on the impact of these factors on recycling behavior. Essentially, the main motivation of this study is to create a general framework that would aid in the development of local e-waste collection policies based on the basic socio-demographic factors that cause variations from region to region in the recycling rate of e-waste.

**Comment 3:**

The introduction provides a through background but lacks a clear statement of the research gap. Clarify the specific limitations of the existing models and how this paper aims to address these limitations. Including a direct statement of the research gap can strengthen the narrative.

**Response 3:**

***This paragraph is added to the Introduction to address the gap.***

While survey-based studies have identified the qualitative and quantitative factors influencing e-waste recycling behavior, a significant gap remains in establishing a framework that incorporates these factors into the planning of the e-waste recycling process. This study seeks to address this gap, specifically by incorporating quantifiable socio-demographic factors into the waste container allocation model, addressing these factors at both macro and micro levels.

**Comment 4:**

The literature review is quite comprehensive, but it could be enriched by adding more recent studies or cases from other countries.

**Response 4:**

**The following studies are added to enrich the literature.**

1. Pakpour AH, Zeidi IM, Emamjomeh MM, Asefzadeh S, Pearson H. Household waste behaviours among a community sample in Iran: An application of the theory of planned behaviour. Waste Manag. 2014 Jun 1;34(6):980–6.
2. Sidique SF, Lupi F, Joshi S V. The effects of behavior and attitudes on drop-off recycling activities. Resour Conserv Recycl. 2010 Jan 1;54(3):163–70.
3. Xu L, Ling M, Lu Y, Shen M. Understanding Household Waste Separation Behaviour: Testing the Roles of Moral, Past Experience, and Perceived Policy Effectiveness within the Theory of Planned Behaviour. Sustainability. 2017 Apr 17;9(4):625.
4. Quynh Vo C, Samuelsen PJ, Leikny Sommerseth H, Wisløff T, Wilsgaard T, Elise Eggen A. Comparing the sociodemographic characteristics of participants and non-participants in the population-based Tromsø Study. BMC Public Health. 2023 Dec 1;23(1).

**Comment 5:**

Authors are requested to add suggested published articles to improve the quality of the work, these are

1. E-waste circularity in India: identifying and overcoming key barriers, Journal of Material Cycles and Waste Management 26 (6), 3928-3945, https://doi.org/10.1007/s10163-024-02050-1.

2. Inconsistencies of e-waste management in developing nations - Facts and plausible solutions, Journal of Environmental Management, 261, 2020, 110234, https://doi.org/10.1016/j.jenvman.2020.110234.

3. Prioritizing Factors Affecting E-Waste Recycling in India: A Framework for Achieving a Circular Economy, Circular Economy and Sustainability, 1-21, <https://doi.org/10.1007/s43615-024-00423-0>.

**Response 5:**

***The suggested studies have been examined.***

In the study titled "*Inconsistencies of e-waste management in developing nations: Facts and plausible solutions*," ten major deficiencies in e-waste management at the strategic level have been identified. In this context, our study does not overlap in terms of scope since it focuses on the factors affecting e-waste recycling as the first-step policies.

In the study titled "*E-waste circularity in India: identifying and overcoming key barriers*," obstacles such as lack of incentives for e-waste recycling in India, customer awareness, and government policies were addressed. Also, in the study titled "*Prioritizing Factors Affecting E-Waste Recycling in India: A Framework for Achieving a Circular Economy*," from a reverse perspective, eleven critical factors affecting e-waste recycling management in India were identified, emphasizing circular economy adoption incentives, government policies, and customer awareness.

Since both of these two studies do not overlap in terms of scope with our study*, it was not deemed necessary to add them to the literature.*

**Comment 6:**

Compare the effectiveness of traditional and green methods with specific case studies or experimental data.

**Response 6:**

First, the approach we propose is preferred not to be called a completely "*green method*" but rather a "*sustainable method."*

In addition, the biggest difference with the *traditional approach* is related to the amount of e-waste expected to be collected. Namely, when the factors affecting recycling are not included in the container assignment model, the amount of waste to be obtained is considerably higher than the amount of waste to be obtained when they are included. This situation has already been mentioned in the results-scenario analysis section, and the relevant data has been given in Table 2.

It can be concluded that the approach, which is thought to be effective on the first-step e-waste recycling decisions of local decision-makers, can also prevent the assignment of extra containers from this perspective and thus prevent unnecessary costs.

***This following statement is added to the paragraph, after Table 2.***

“So, this proposed approach, deemed effective in the initial phase of e-waste recycling decisions by local authorities, can prevent the allocation of unnecessary containers and associated costs.”

**Comment 7:**

Add research methodology flow chart of the study.

**Response 7:**

***The flow chart of the research methodology has already been added as***

“**Figure 3.** The general flow of the research methodology.” The name is changed.

**Comment 8:**

Highlight the potential challenges in implementing the proposed model in real-world scenarios, such as data availability or political constraints.

**Response 8:**

Since the proposed model takes into account quantitative values related to e-waste recycling behavior and is not survey-based, accessing this data is relatively easier than relative factors such as environmental awareness, habits, convenience, etc. In this context, there are no restrictions in accessing and using the data. Regarding the model application, it has been emphasized before that working in a joint project with the local government would yield more realistic results and that this is a weakness of the study.

**Comment 9:**

Write theoretical and practical implications of the work.

**Response 9:**

**The following paragraphs are added to the Conclusion Section for theoretical and practical implications.**

Examining the theoretical framework, it becomes evident that while the model primarily addresses the assignment of waste containers, the findings suggest that the variables integrated into the proposed model have minimal influence on the quantity of containers deployed. The outcome aligns with prior expectations, given that the model incorporates the requirement of allocating a minimum of one container to each region. The variation in the number of containers appears to be primarily attributed to the adjustments made in the coverage distance incorporated within the model. The primary contribution of the proposed model lies in its ability to ascertain the quantity of waste that is expected to be collected which varies in regions with high and low e-waste production potential.

From a practical point of view, the proposed model's generic structure allows for its research findings to be applicable beyond a single region. This versatility enables policymakers in various local governments to incorporate both macro and micro variables, facilitating the development of first-step e-waste recycling policies. Ultimately, this approach aims to foster the establishment of more sustainable practices in e-waste recycling. Moreover, collaborative research efforts with local authorities are set to lead to more accurate outcomes.

**Comment 10:**

The conclusion could be more concise. Focus on the key findings and the specific improvements your method offers over existing models. Refrain from repeating details already covered in previous sections.

**Response 10:**

Some paragraphs marked in the revised manuscript in the conclusion section were removed to simplify the text. In addition, the paragraphs in Comment 9 and the sections requested by the other referee were added, and the Discussion and Conclusion sections were rearranged.

**Reviewer #2 and Reviewer #3:**

The Editor indicated that since they are concerned about the possibility that an AI tool was used to create the review, ***I do not need to reply to the remarks made by reviewers 2 and 3.***

**Reviewer #4:**

Dear Authors,

Your submission, "A Case Study of Transferring the Effect of Demographic Factors on E-Waste Recycling to the Waste Container Assignment Model," has been received and reviewed. An important and pertinent topic in waste management research is the study's innovative method of incorporating demographic variables into e-waste container assignment models.

My detailed comments and suggestions are in the attached file.

**Comment 1:**

Among these factors, it is obvious that the income and educational levels of electronic consumers, as well as the age and population size distribution in the surrounding area, are all significant.

**Response 1:**

In place of the abovementioned phrase, the following phrase is added to the manuscript:

A significant portion of the studies on recycling behavior state that socio-demographic characteristics have a significant effect on recycling behavior(1–4), while others claim that these factors play only a minimal role (5).

**Comment 2:**

sociodemographic characteristics

**Response 2:**

An explanation for sociodemographic characteristics has been added at the beginning of the paragraph with a reference (6).

Socio-demographic characteristics encompass a range of social and demographic factors, which are often measured by an individual's age, level of education, occupation, and income.

**Comment 3:**

The main purpose of this study is to create a generic assignment model based on the factors affecting electronic waste generation.

**Response 3:**

The relevant sentence was removed from the materials and methods section and combined with a similar sentence in the introduction.

**Comment 4:**

**Figure 2.** Country-based differences affecting WEEE consumption in the macro sense and Socio-economic/demographic factors affecting WEEE behavior in the micro sense

**Response 4:**

Figure 2 is linked to the body text of the manuscript previous paragraph on page 4.

**Comment 5:**

**Figure 3.** The general flow of the proposed approach

**Response 5:**

Figure 3 has been revised by removing the statement "factors affecting e-waste behavior" in step 1.

**Comment 6:**

the televisions /monitors and informatics-telecommunication equipment

**Response 6:**

*The following paragraph was added to explain this research is only for e-waste from the televisions/monitors and informatics-telecommunication equipment category.*

“E-waste encompasses an extensive variety of products. As previously stated, e-waste is categorized into six classes in accordance with the European Union WEEE Directive (Fig. 1). This study focuses on the collection of e-waste categories ranked second (displays) and sixth (telecommunication equipment). The application is restricted to these two categories because items like monitors, tablets, mobile phones, and headphones are of sizes and weights portable by household and the container capacities designated in the model are appropriate for this context. For instance, items like refrigerators in category 1 and washing machines in category 4 are not suitable for households to transport to the nearest disposal container. Once more, light bulbs in category 3 were excluded from the model due to their hazardous contents that could be released upon breakage. Category 5 was also excluded from this model due to its composition mostly consisting of waste that is likely to surpass the container's capacity."

**Comment 7:**

potential locations

**Response 7:**

*As you stated, regarding potential locations, the old paragraph has been completely deleted and the following paragraph, which is more explanatory, has been added.*

“The potential number of e-waste containers is calculated by dividing the total budget by the unit container price, utilizing the budget allocated by the local government for recycling activities. The maximum number of containers defined in the manuscript is 23. Figure 4 illustrates the distribution of candidate container points across eight neighborhoods. The number of containers assigned to each neighborhood is detailed in Table in S3 Table.”

**Comment 8:**

**Discussion and Conclusions**

**Response 8:**

*The following two paragraphs have been added to the discussion section for explanatory purposes.*

“As previously stated, many studies have demonstrated that e-waste recycling behaviors can be analyzed by accounting for a wide range of socio-economic and demographic factors, including income, gender, education level, age, environmental consciousness, habits, convenience, etc. The results reveal that the distinct characteristics of each country might influence individuals' participation in e-waste collection and recycling efforts in various manners.

Even though numerous studies have discussed these factors, none of them have directly included them in the waste container assignment model. The regional ratios derived from these factors have been integrated into the e-waste consumption capacity constraint through a harmonic mean. So, the proposed approach distinguishes itself from existing literature by incorporating factors influencing e-waste recycling into the e-waste container allocation model, addressing these factors at both macro and micro levels.”

**References**

1.	Saphores J. Household willingness to recycle electronic waste: an application to California. Environ Behav [Internet]. 2006 Mar [cited 2023 Sep 7];38(2):183–208. Available from: https://journals.sagepub.com/doi/abs/10.1177/0013916505279045

2.	Pakpour AH, Zeidi IM, Emamjomeh MM, Asefzadeh S, Pearson H. Household waste behaviours among a community sample in Iran: An application of the theory of planned behaviour. Waste Manag. 2014 Jun 1;34(6):980–6.

3.	Sidique SF, Lupi F, Joshi S V. The effects of behavior and attitudes on drop-off recycling activities. Resour Conserv Recycl. 2010 Jan 1;54(3):163–70.

4.	Xu L, Ling M, Lu Y, Shen M. Understanding Household Waste Separation Behaviour: Testing the Roles of Moral, Past Experience, and Perceived Policy Effectiveness within the Theory of Planned Behaviour. Sustainability [Internet]. 2017 Apr 17;9(4):625. Available from: https://www.mdpi.com/2071-1050/9/4/625/htm

5.	Saphores J, Ogunseitan O, Shapiro A. Willingness to engage in a pro-environmental behavior: An analysis of e-waste recycling based on a national survey of U.S. households. Resour Conserv Recycl. 2012 Mar 1;60:49–63.

6.	Quynh Vo C, Samuelsen PJ, Leikny Sommerseth H, Wisløff T, Wilsgaard T, Elise Eggen A. Comparing the sociodemographic characteristics of participants and non-participants in the population-based Tromsø Study. BMC Public Health. 2023 Dec 1;23(1).
