# Supplementary material for: A case study of transferring the effect of demographic factors on e-waste recycling to the waste container assignment model
Source: PLoS One. 2025 Aug 25;20(8):e0315695. doi: 10.1371/journal.pone.0315695 (PMC12377600; doi:10.1371/journal.pone.0315695)
Supplement: Attachment — Submitted filename: Reviewer Feedback on Manuscript.docx [file pone.0315695.s012.docx]

**Reviewer Feedback on Manuscript**

**General Assessment**

This study introduces an innovative approach to optimizing e-waste container placement by integrating sociodemographic factors (population, income, education, and age distribution) into a mathematical assignment model. By incorporating both micro-level (regional) and macro-level (country-based) elements, the research addresses a notable gap in municipal e-waste management decision-making. The manuscript is well-structured, with clear sections on motivation, literature review, methodology, and case study. However, some areas require clarification, enhanced methodological rigor, and expanded discussion to strengthen its overall contribution.

**Strengths**

1. Novelty & Contribution: The integration of sociodemographic factors into an e-waste container assignment model is a significant innovation, addressing a key research gap. The model provides a versatile framework applicable across various regions and countries, increasing its practical relevance.
2. Methodological Rigor: The use of the harmonic mean to balance extreme values in sociodemographic data is well-justified and mathematically sound. The mixed-integer linear programming (MILP) formulation is clearly explained, with well-defined constraints and decision variables.
3. Practical Implications: The case study illustrates how local governments can leverage the model to optimize e-waste collection policies. The scenario analysis (varying coverage distance) offers valuable insights into the trade-offs between accessibility and collection efficiency.

**Weaknesses & Suggested Improvements**

1. Literature Review & Theoretical Foundation

i. Gap Justification: The claim that *"no research has incorporated demographic characteristics into assignment models"* needs refinement. Some facility location models in waste management implicitly consider population density or income. A brief discussion of relevant urban waste collection optimization studies would strengthen the study’s positioning.
ii. Behavioral Factors: While the paper references study on recycling behavior, it does not fully explain why specific factors (income, education, age) were chosen over others (e.g., environmental awareness, incentives). A stronger theoretical foundation, such as the Theory of Planned Behavior, would be beneficial.

2**. Model Assumptions & Limitations**

- Container Capacity & Types: The assumption that containers are only suitable for categories 2 & 6 (displays, telecom) is reasonable but restrictive. Can the model be extended to accommodate additional categories with modified constraints?

i. Distance Sensitivity: The model assumes a fixed coverage distance (Dc), yet willingness to recycle likely declines nonlinearly with distance. Exploring a distance decay function (e.g., exponential decay) could improve realism.
ii. Dynamic vs. Static Model: The current model is static (one-time placement). Could a dynamic version be developed to account for seasonal variations in e-waste generation?

3**. Case Study & Data**

i. Data Sources: The case study relies on secondary data (e.g., e-waste per household in Turkey). Were any primary surveys conducted to validate assumptions (e.g., actual recycling behavior in the studied region)?
ii. Sensitivity Analysis: The harmonic mean reduces extreme values, but how sensitive are results to weighting schemes (equal vs. unequal factor weights)?
iii. Comparison with Baseline: The study contrasts "factors included" vs. "factors excluded" but lacks benchmarking against existing municipal practices (e.g., uniform container distribution).

**4. Results & Discussion**

i. Interpretation of Findings: The tenfold difference in e-waste collection estimates (Table 1) is striking. Does this suggest that current municipal policies significantly overestimate collection rates?
ii. The reduction in container numbers with increased Dc (Fig 5 vs. Fig 6) highlights trade-offs between accessibility and cost. Could this be quantified via a cost-benefit analysis? Policy Implications:
iii. The discussion should elaborate on how municipalities can implement the model, addressing key factors such as budget allocation and stakeholder engagement.

5. Clarity & Presentation

i. Equation Formatting: Some equations (e.g., Equation 7) are not well-formatted. Ensure consistency in notation (subscripts/superscripts).
ii. Figures/Tables:
    a. Fig 1 (WEEE categories) should be included in the main text rather than just being referenced.
    b. Table 3: The assignment matrix is challenging to interpret. A heatmap visualization might improve clarity.

**Recommendation**

This paper makes a valuable contribution to e-waste management by integrating sociodemographic factors into container assignment. However, revisions are needed to:

i. Strengthen the literature review (improve positioning relative to prior work).
ii. Enhance methodological transparency (sensitivity analysis, dynamic considerations).
iii. Improve case study validation (primary data, benchmarking).
iv. Clarify policy implications (practical implementation strategies).

**Decision: Minor Revisions required before acceptance.**
