# Supplementary material for: A case study of transferring the effect of demographic factors on e-waste recycling to the waste container assignment model
Source: PLoS One. 2025 Aug 25;20(8):e0315695. doi: 10.1371/journal.pone.0315695 (PMC12377600; doi:10.1371/journal.pone.0315695)
Supplement: Attachment — Submitted filename: Response to Reviewers 2.docx [file pone.0315695.s013.docx]

**Response to Reviewers**

**Additional Editor Comments:**

Comments from the editorial office: Upon internal evaluation of the reviews provided, we kindly request you to disregard the reviewer report provided by Reviewer 1. No amendments are required in response to reviewer 3’s comments’

*As it is stated in “Additional Editor Comments,” we disregarded the reviewer report provided by Reviewer 1 and 3.* ***We solely based our considerations and responses on the editor's reviewer report.***

**Weaknesses & Suggested Improvements**

1. Literature Review & Theoretical Foundation

**Comment 1:**

i. **Gap Justification:** The claim that *"no research has incorporated demographic characteristics into assignment models"* needs refinement. Some facility location models in waste management implicitly consider population density or income. A brief discussion of relevant urban waste collection optimization studies would strengthen the study’s positioning.

**Response 1:**

As suggested, to strengthen the position of the study, *the following text is added to the Literature section, and the Literature section is also restructured.*

“*[1] developed a mixed integer linear programming (MILP) model that allows a single waste collector to be allocated to several communities within a neighborhood, considering both the total waste separation volume of collectors and their proximity to the neighborhood. [2] has proposed a two objective mixed integer nonlinear model for scenarios where consumers deposit their e-waste at a collection center and receive a fixed financial incentive or where e-waste is collected at their doorstep without receiving any financial incentive. The objectives are to minimize the collection costs and maximize the amount of WEEE collected. For the WEEE drop-off case, two factors, namely incentive and ease of disposal (i.e., distance between the customer node and the collection center), are considered.*

*In the MILP model constructed in [3] the optimum number and location of collection points, collection centers, and recycling centers were determined to minimize the overall costs, which include the fixed costs associated with e-waste facilities and the transportation costs between these facilities. The study focused on minimizing e-waste costs [4], including collection, transportation, and processing, while aiming to maximize resource recovery and reduce environmental impact through a multi-objective MILP model. However, none of the above models has accounted for sociodemographic factors affecting e-waste demand in the assignment model.*

*A binary logistic regression analysis was done using survey results to see how factors like income, age, education, knowledge, habits, traditions, incentive payments, and behaviors affect people's willingness to recycle mobile phones [5]. Furthermore, the literature includes studies that incorporate financial incentives into the assignment model as a factor influencing e-waste consumption [2,6]. However, no research has identified sociodemographic factors affecting e-waste demand as a direct component in the container allocation model.”*

“*In summary, despite numerous studies addressing the determinant factors, none have concentrated on the impact of these factors on recycling behavior*.” has been deleted and

“*In the current literature, there is a lack of research that directly integrates socio-demographic characteristics affecting e-waste consumption within the container allocation model.”* has been added to express its contribution to the literature more clearly in page 6, at the end of “Literature” section.

**Comment 2:**

ii. Behavioral Factors: While the paper references study on recycling behavior, it does not fully explain why specific factors (income, education, age) were chosen over others (e.g., environmental awareness, incentives). A stronger theoretical foundation, such as the Theory of Planned Behavior, would be beneficial.

**Response 2:**

To fully explain why certain factors (e.g., income, education, age) are preferred over others (e.g., environmental awareness, incentives), the following section containing some of the behavioral research on recycling using the Theory of Planned Behavior has been added to the section titled "Factors affecting e-waste recycling."

“*Recycling necessitates individual effort due to the need to separate, prepare, and store e-waste. Consequently, the decision to recycle is complex and involves various factors.*

*The Theory of Planned Behavior (TPB) is one of the most popular models preferred in behavioral research on recycling and has been used in many studies on the determinants of e-waste recycling* [7–11]*.* *In its simplest form, TPB explains behavioral intention as a function of three components: attitude, subjective norm, and perceived behavioral control. In their highly cited study,* [8] *included the three main components of TPB, as well as socio-demographic factors, the degree of awareness of the problem, and the environmental situation of Brazil. Previous studies have shown that, apart from the behavioral aspects of consumers' e-waste recycling behavior, there is also a relationship between demographic and socioeconomic variables such as household gender, age, education, and income* [12,13]*.*

*Another reason for preferring these factors for e-waste recycling in this study is their ability to be included in the mathematical model with quantitative values.”*

2**. Model Assumptions & Limitations**

**Comment 3:**

- Container Capacity & Types: The assumption that containers are only suitable for categories 2 & 6 (displays, telecom) is reasonable but restrictive. Can the model be extended to accommodate additional categories with modified constraints?

**Response 3:**

Although e-waste encompasses a wide variety of types, including large household appliances, computers, mobile phones, televisions, medical devices, sports equipment, toys, etc., the reverse logistics model discussed in this study covers e-waste that the consumer brings to the collection point with their means.

The legal frameworks, in most European countries that require consumers to transport recyclable waste with their vehicles to large collection areas are not yet implemented in most Asian countries.

In Turkey, the general practice adopted by local governments is to place boxes the size of garbage containers at designated points and have end consumers bring their e-waste to these points. Therefore, in practice, Categories 2 and 6 were considered as e-waste types. *This situation has already been explained in the manuscript, on page 14, paragraph 1.*

*But* ***to answer your question****,* yes, the right-hand side value of the capacity constraint (Eq. 6) of the mathematical model can be updated by considering different sizes of e-waste containers. *In this context, the following sentence has been added in response to Comment 3 in section titled as “Capacity of an e-waste container (S)” at page 16.*

*“However, larger or smaller-sized e-waste containers could be included in the process by changing the right-hand side value in the capacity constraint* *(Eq. 6) in the model.”*

**Comment 4:**

i. Distance Sensitivity: The model assumes a fixed coverage distance (Dc), yet willingness to recycle likely declines nonlinearly with distance. Exploring a distance decay function (e.g., exponential decay) could improve realism.

**Response 4:**

Of course, the demand generated at a node decreases exponentially with distance from the facility [14] but since we formulated our assignment problem as **a mixed linear integer program**, that is, as a MILP, we examined the effect of discrete values of Dc on the model results in Table 2 instead of integrating it into the model exponentially, etc.

Nevertheless, *the following statement is added to the suggestions for future studies in the “****Discussion and conclusions”*** *section.*

*“Since the demand generated at a node decreases exponentially with distance from the facility, a non-linear model might be more suitable for the assignment problem instead of using discrete values of Dc.”*

**Comment 5:**

ii. Dynamic vs. Static Model: The current model is static (one-time placement). Could a dynamic version be developed to account for seasonal variations in e-waste generation?

**Response 5:**

The socio-demographic factors that constitute the main motivation for the establishment of the model and affect the amount of e-waste to be consumed are factors that do not change rapidly over time. Seasonal changes such as weather conditions are not taken into account for this model. The proposed MILP model is deterministic and static in its current form, and changes in socio-demographic factors over time in the region under consideration can only be reflected in the model as parameter changes.

3**. Case Study & Data**

**Comment 6:**

i. Data Sources: The case study relies on secondary data (e.g., e-waste per household in Turkey). Were any primary surveys conducted to validate assumptions (e.g., actual recycling behavior in the studied region)?

**Response 6:**

Most of the research on this subject is survey-oriented. Another feature that distinguishes this study from other studies in the literature is that it is not based on a survey. Survey studies are, of course, valuable, but the socio-demographic factors used here (income and education status, age, and population rate) are information that local governments can currently collect in different ways. In order to obtain these, there is no need to conduct a survey, as is the case with subjective factors such as attitude, behavior, and desire, etc. In this study, these determinants were included in the assignment model in a way that also predicts e-waste demand to obtain both a practical and rapid result.

**Comment 7:**

ii. Sensitivity Analysis: The harmonic mean reduces extreme values, but how sensitive are results to weighting schemes (equal vs. unequal factor weights)?

**Response 7:**

In this study, it is assumed that all sociodemographic factors affect the amount of e-waste production equally. However, MCDM techniques can be applied to determine which of these factors affect the process to what extent. *This situation was already suggested in the "results and discussions" section for subsequent studies. Therefore, no additions are made to the manuscript.*

**Comment 8:**

iii. Comparison with Baseline: The study contrasts "factors included" vs. "factors excluded" but lacks benchmarking against existing municipal practices (e.g., uniform container distribution).

**Response 8:**

*The following paragraph titled "****Comparison with the current situation****" has been added to the "Results" section.*

*“The municipality, where the case study was carried out, placed seven-compartment containers for paper, plastic, glass, metal, vegetable oil, small electronic goods, and battery waste at 24 points within the district borders approximately three years ago. The municipality performance report stated that the average annual e-waste amount for recycling over the last three years was 500 kg [15]. If the difference between the model's values and the actual values is interpreted, it should first be noted that the model's values, like the total budget and container cost, are entirely hypothetical. At the same time, the capacity of the container is the main factor leading to the high e-waste values predicted by the model and expected for collection. The containers proposed in the study are only for the purpose of collecting e-waste, and their capacity is considered to be approximately 4.5 m³/1500 kg. It also focused on the collection of WEEE in the 2nd and 6th categories, which include medium-sized and voluminous e-waste such as televisions, monitors, and tablets. However, the containers currently used by the municipality consist of seven small compartments, and only one of these compartments (approximately 0.6 m³/150 kg) is reserved for e-waste. Although e-waste collection activities are partially carried out in the region covered by the study, they are inadequate, as can be understood by the annual average collected values.*

*The model suggested in the study looks from a different point and predicts how much e-waste could be collected if containers with the suggested size are set up at the locations chosen by the model.”*

**4. Results & Discussion**

**Comment 9:**

i. Interpretation of Findings: The tenfold difference in e-waste collection estimates (Table 1) is striking. Does this suggest that current municipal policies significantly overestimate collection rates?

**Response 9:**

The “factors not included” values shown in Table 1 should not be considered as the current municipality’s targets for e-waste collection. These values represent the values that would be obtained if the proposed model was run without considering the demographic factors that affect the amount of e-waste consumption. In other words, they reflect the result of a sensitivity analysis showing the effect of the factors on the model.

*To avoid misunderstanding, the following statement has been added below Table 1.*

*“The values labeled as "factors not included" in Table 1 represent the values that would be obtained if the proposed model was run without considering the demographic factors that affect the amount of e-waste consumption; they reflect the effect of the factors on the model.”*

**Comment 10:**

ii. The reduction in container numbers with increased Dc (Fig 5 vs. Fig 6) highlights trade-offs between accessibility and cost. Could this be quantified via a cost-benefit analysis?

**Response 10:**

As the survey-based studies show, the desire for recycling decreases as the distance between the consumer and the e-waste container increases. The results of the analysis we conducted also support this situation. Herein, the benefit to be obtained is the collection of more e-waste, while the cost will be the placement of more containers and the costs of collecting them. However, since we do not have clear financial data regarding this situation and definite information about the financial gain of recycling, applying a quantitative cost-benefit analysis to this case study may not yield very effective results. Perhaps, in a future study, more data can be obtained through a joint project with the municipality, and the integration of the cost-benefit analysis into the assignment model can be tried. In this study, instead of selecting the coverage distance as a large or small value, the solution of determining the most appropriate distance by conducting various trials over different coverage distances is suggested to decision makers.

**Comment 11:**

Policy Implications:

iii. The discussion should elaborate on how municipalities can implement the model, addressing key factors such as budget allocation and stakeholder engagement.

**Response 11:**

As stated before, although local governments attach importance to waste collection in practice, they often prefer intuitive and palliative solutions instead of systematic and permanent solutions. As a general practice, points where crowded passages are intense, such as markets, parks, and ATMs, are intuitively preferred for placing waste recycling containers. The municipality where the application is carried out has placed seven-compartment containers for paper, plastic, glass, metal, vegetable oil, small electronic goods, and battery waste at 24 points within the district borders. However, separate containers focused solely on e-waste collection are not used. Moreover, since there is only one compartment allocated for e-waste, it only has a capacity to accommodate a very limited number of e-waste items, such as headphones, TV remote controls, speakers, mobile phones, or small computer parts. This situation also seriously affects the e-waste collection potential.

This study proposes a generic model that assigns the appropriate number of e-waste containers to the optimum points, considering the most appropriate coverage distance, according to the socio-demographic profile of local governments.

*However, the objective function of the mathematical model created in this study is not focused on cost minimization but rather aims to maximize the indirect benefit to be obtained by increasing the e-waste amount to be collected. In this context, the model does not create a direct budgetary consequence for the decision makers.*

*The following paragraph has been added to address the deficiencies in comment 11 regarding "explaining in detail how municipalities can implement the model."*

*“In practice, it is seen that in terms of waste management, local governments often prefer intuitive and palliative solutions rather than systematic and permanent solutions. In general, local governments intuitively prefer to place waste recycling containers in crowded passageways like markets, parks, and ATMs. For instance, the municipality where the case study is carried out has placed seven-compartment containers for paper, plastic, glass, metal, vegetable oil, small electronic goods, and battery waste at 24 points within the district borders. However, separate containers focused solely on e-waste collection are not used. Moreover, since there is only one compartment allocated for e-waste, it only has a capacity to accommodate a very limited number of e-waste items, such as headphones, TV remote controls, speakers, mobile phones, or small computer parts. This situation also seriously affects the e-waste collection potential.*

*This study proposes a generic model that assigns the appropriate number of e-waste containers to the optimum points, considering the most appropriate coverage distance, according to the socio-demographic profile of local governments.”*

5. Clarity & Presentation

**Comment 12:**

i. Equation Formatting: Some equations (e.g., Equation 7) are not well-formatted. Ensure consistency in notation (subscripts/superscripts).

**Response 12:**

*All equations in the "Model formulation" section on page 10 have been reviewed, we have also removed and edited unnecessary equations from the "Constitution of parameters" section.*

*Equation 7 has been edited as follows to ensure consistency in notation.*

**Comment 13:**

ii. Figures/Tables:
    a. Fig 1 (WEEE categories) should be included in the main text rather than just being referenced.

**Response 13/a:**

*The following paragraph is added to include Figure 1 (WEEE categories) in the main text rather than using it as a reference only.*

Large household appliances, computers, cell phones, televisions, medical equipment, sports gear, toys, and other discarded electronic devices are all considered e-waste. However, the model in this study includes e-waste that the consumer transports to the collection points on their own as categories 2 and 6, colored red in Fig. 1.

b. Table 3: The assignment matrix is challenging to interpret. A heatmap visualization might improve clarity.

**Response 13/b:**

*As suggested, a heatmap view has been added to the assignment matrix in Table 3 to improve clarity and visibility.*

**Table 3.** (a) Assignment results for maximum coverage distance of 1000 m (b) Assignment results for maximum coverage distance of 2000 m

|  | ***(a) Dc=1000 m*** | | | | | | | | ***(b) Dc= 2000 m*** | | | | | | | | | |
| --- | --- | --- | --- | --- | --- | --- | --- | --- | --- | --- | --- | --- | --- | --- | --- | --- | --- | --- |
|  | **Regions** | | | | | | | | | | | | | | | | | |
| ***Z=2956.5*** |  |  |  |  |  |  |  |  | ***Z=3972*** |  |  |  |  |  |  |  |  |  |
| ***Candidate Points*** | **1** | **2** | **3** | **4** | **5** | **6** | **7** | **8** | ***Candidate Points*** | **1** | **2** | **3** | **4** | **5** | **6** | **7** | **8** |  |
| *1* | 1 | - | - | - | - | - | - | - | *1* | 1 | - | - | - | - | - | - | - |  |
| *2* | - | - | - | - | - | - | - | - | *2* | - | 1 | - | - | 1 | - | - | - |  |
| *3* | - | - | - | - | - | - | - | - | *3* | 1 | - |  | - | - | - | - | - |  |
| *4* | - | - | - | - | 1 | - | - | - | *4* | 1 | 1 | - | 1 | 1 | 1 | - | - |  |
| *5* | - | 1 | - | - | - | - | - | - | *5* | - | - | - | - | - | - | - | - |  |
| *6* | - | 1 | - | - | - | - | - | - | *6* | - | - | - | - | - | - | - | - |  |
| *7* | - | 1 | - | - | - | - | - | - | *7* | - | - | - | - | - | 1 | - | - |  |
| *8* | - | 1 | - | - | - | - | - | - | *8* | - | - | - | - | - | - | 1 | - |  |
| *9* | - | - | 1 | - | - | - | - | - | *9* | - | - | 1 | - | - | - | - | - |  |
| *10* | - | - | - | - | - | - | - | - | *10* | 1 | - | 1 | - | - | - | - | - |  |
| *11* | - | - | - | - | - | - | - | - | *11* | - | - | 1 | - | 1 | - | - | - |  |
| *12* | - | - | - | - | 1 | - | - | - | *12* | - | - | - | 1 | - | - | - | - |  |
| *13* | - | - | - | 1 | - | - | - | - | *13* | - | - | - | 1 | - | - | - | - |  |
| *14* | - | - | - | 1 | - | 1 | - | - | *14* | - | - | - | - | - | - | - | - |  |
| *15* | - | - | - | - | - | - | - | - | *15* | - | 1 | - | - | - | - |  | - |  |
| *16* | - | - | - | 1 | - | - | - | - | *16* | - | - | - | - | - | - | - | - |  |
| *17* | - | - | - | - | - | - | - | - | *17* | - | - | - | - | - | - | - | - |  |
| *18* | - | - | - | - | - | - | - | - | *18* | - | - | - | - | - | - | 1 | 1 |  |
| *19* | - | - | - | - | - | 1 | - | - | *19* | - | 1 | - | - | - | - | - | - |  |
| *20* | - | - | - | - | - | - | 1 | - | *20* | - | - | - | - | - | - | - | 1 |  |
| *21* | - | - | - | - | 1 | - | - | - | *21* | - | - | - | - | - | - | - | - |  |
| *22* | - | - | - | - | - | - | 1 | 1 | *22* | - | - | - | - | - | - | - | - |  |
| *23* | - | - | - | - | - | - | - | 1 | *23* | - | - | - | - | - | - | - | - |  |
| ***CN*** | **1** | **4** | **1** | **3** | **3** | **2** | **2** | **2** | ***CN*** | **4** | **4** | **3** | **3** | **3** | **2** | **2** | **2** |  |

**Recommendation**

This paper makes a valuable contribution to e-waste management by integrating sociodemographic factors into container assignment. However, revisions are needed to:

i. Strengthen the literature review (improve positioning relative to prior work).
ii. Enhance methodological transparency (sensitivity analysis, dynamic considerations).
iii. Improve case study validation (primary data, benchmarking).
iv. Clarify policy implications (practical implementation strategies).

**Decision: Minor Revisions required before acceptance.**

*Dear Editor,*

*Initially, we would like to express our gratitude for your valuable contributions to the research. We tried to produce the most appropriate solutions to your criticisms by answering all the questions step by step. We hope it was adequate.*

*Best regards.*

REFERENCES

1.	Lin HY. A mathematical programming model integrating waste pickers in urban area recycling practice. City and Environment Interactions. 2024;24: 100158. doi:10.1016/J.CACINT.2024.100158

2.	Koshta N, Patra S, Singh SP. A location-allocation model for E-waste acquisition from households. Journal of Cleaner Production. 2024;440: 140802. doi:10.1016/J.JCLEPRO.2024.140802

3.	Chouksey A, Agrawal M, Jha R. Planning of three-level facilities network for collection and recycling of E-waste: A MILP-based modelling approach. Materials Today: Proceedings. 2024;112: 54–59. doi:10.1016/J.MATPR.2023.08.026

4.	Ali W. Optimizing the e-waste management in India: a sustainable mathematical modeling approach to circular economy. Quality and Quantity. 2025; 1–32. doi:10.1007/S11135-025-02176-W/FIGURES/7

5.	Cai K, Wang L, Ke J, He X, Song Q, Hu J, et al. Differences and determinants for polluted area, urban and rural residents’ willingness to hand over and pay for waste mobile phone recycling: Evidence from China. Waste Management. 2023;157: 290–300. doi:10.1016/J.WASMAN.2022.12.032

6.	Aras N, Aksen D. Locating collection centers for distance- and incentive-dependent returns. International Journal of Production Economics. 2008;111: 316–333. doi:10.1016/J.IJPE.2007.01.015

7.	Kumar A. Exploring young adults’ e-waste recycling behaviour using an extended theory of planned behaviour model: A cross-cultural study. Resources, Conservation and Recycling. 2019;141: 378–389. doi:10.1016/J.RESCONREC.2018.10.013

8.	Echegaray F, Hansstein F. Assessing the intention-behavior gap in electronic waste recycling: the case of Brazil. Journal of Cleaner Production. 2017;142. Available: https://www.sciencedirect.com/science/article/pii/S0959652616305285

9.	Wang Z, Guo D, Wang X. Determinants of residents’e-waste recycling behaviour intentions: evidence from China. Journal of Cleaner Production. 2016 [cited 12 Feb 2024]. Available: https://www.sciencedirect.com/science/article/pii/S0959652616310514

10.	Aboelmaged M. E-waste recycling behaviour: An integration of recycling habits into the theory of planned behaviour. Journal of Cleaner Production. 2021 [cited 3 Nov 2022]. Available: https://www.sciencedirect.com/science/article/pii/S095965262034227X

11.	Nguyen HTT, Hung RJ, Lee CH, Nguyen HTT. Determinants of Residents’ E-Waste Recycling Behavioral Intention: A Case Study from Vietnam. Sustainability. 2018;11: 164. doi:10.3390/SU11010164

12.	Saphores J. Household willingness to recycle electronic waste: an application to California. Environment and Behavior. 2006;38: 183–208. doi:10.1177/0013916505279045

13.	Sidique SF, Lupi F, Joshi S V. The effects of behavior and attitudes on drop-off recycling activities. Resources, Conservation and Recycling. 2010;54: 163–170. doi:10.1016/J.RESCONREC.2009.07.012

14.	Berman O, Drezner Z. Location of congested capacitated facilities with distance-sensitive demand. IIE Transactions. 2006;38: 213–221. doi:10.1080/07408170500288190

15.	Yakutiye Municipality. Yakutiye Municipality 2024 Performance Report. 2024.
